# Population substructure in Finland and Sweden revealed by the use of spatial coordinates and a small number of unlinked autosomal SNPs

**DOI:** 10.1186/1471-2156-9-54

**Published:** 2008-08-19

**Authors:** Ulf Hannelius, Elina Salmela, Tuuli Lappalainen, Gilles Guillot, Cecilia M Lindgren, Ulrika von Döbeln, Päivi Lahermo, Juha Kere

**Affiliations:** 1 Department of Biosciences and Nutrition, Karolinska Institutet, 14157 Huddinge, Sweden; 2 Department of Medical Genetics, University of Helsinki, Helsinki, Finland; 3 Finnish Genome Center, Institute for Molecular Medicine Finland, University of Helsinki, Helsinki, Finland; 4 Centre for Ecological and Evolutionary Synthesis, Department of Biology, University of Oslo, Norway; 5 Wellcome Trust Centre for Human Genetics, Oxford University, Oxford, UK; 6 Centre for Inherited Metabolic Diseases, Karolinska University Hospital, Stockholm, Sweden; 7 Clinical Research Centre, Karolinska University Hospital, Stockholm, Sweden

## Abstract

**Background:**

Despite several thousands of years of close contacts, there are genetic differences between the neighbouring countries of Finland and Sweden. Within Finland, signs of an east-west duality have been observed, whereas the population structure within Sweden has been suggested to be more subtle. With a fine-scale substructure like this, inferring the cluster membership of individuals requires a large number of markers. However, some studies have suggested that this number could be reduced if the individual spatial coordinates are taken into account in the analysis.

**Results:**

We genotyped 34 unlinked autosomal single nucleotide polymorphisms (SNPs), originally designed for zygosity testing, from 2044 samples from Sweden and 657 samples from Finland, and 30 short tandem repeats (STRs) from 465 Finnish samples. We saw significant population structure within Finland but not between the countries or within Sweden, and isolation by distance within Finland and between the countries. In Sweden, we found a deficit of heterozygotes that we could explain by simulation studies to be due to both a small non-random genotyping error and hidden substructure caused by immigration. Geneland, a model-based Bayesian clustering algorithm, clustered the individuals into groups that corresponded to Sweden and Eastern and Western Finland when spatial coordinates were used, whereas in the absence of spatial information, only one cluster was inferred.

**Conclusion:**

We show that the power to cluster individuals based on their genetic similarity is increased when including information about the spatial coordinates. We also demonstrate the importance of estimating the size and effect of genotyping error in population genetics in order to strengthen the validity of the results.

## Background

The neighbouring countries of Sweden and Finland represent two modern societies with a population history of about 12,000 years and several millennia of close contacts [1]. Due to the geographical and political situation, the countries have been shaped differently by epidemics, wars and migratory waves [2]. The northern and eastern parts of Finland remained mostly uninhabited until the 16^th ^century, and even after that the population size remained small. This has led to extensive genetic drift, pronounced differences between Eastern and Western Finns observed in the Y-chromosomal as well as autosomal variation, and local or regional enrichment of several monogenic diseases in Finns [3-7], (Salmela et al. submitted). The genetic variation of the Swedish population appears clinal in Y-chromosomal and mtDNA analyses of the same sample set used in this study (Lappalainen et al. submitted), as well as in a previous Y-chromosomal study [8]; however, local genetic isolates have been detected in the northern part of Sweden [9].

During the past few years, it has been shown that individuals can be clustered based on genetic similarity, and that these clusters correspond closely to ancestral place of origin [10-12]. It has been estimated that to predict the ancestry of individuals, up to a thousand random single nucleotide polymorphisms (SNPs) or short tandem repeats (STRs) might be needed [13]. By using markers that exhibit large differences in allele frequency between the populations of interest, this number can be reduced [14-16]. Still, such ancestry informative markers (AIMs) are very dependent on the populations used for defining them and may be too specific when used for identifying fine-scale structure [16]. Interestingly, a recent study was able to accurately predict ancestral continent of origin of individuals from two independent data sets by using only a small number of arbitrarily chosen SNPs from the International HapMap Project [17]. The authors concluded, however, that the amount of genotype data would have to be increased in order to make predictions of more fine-scale geographic structures.

The aim of this study was to investigate if the known genetic substructures could be identified within Finland and Sweden by using 34 unlinked autosomal SNPs originally designed for zygosity testing [18]. To compare two different kinds of marker sets and to gain further resolution of the population genetic structure within Finland, we genotyped 30 STRs on a subset of the Finnish samples. Based on the SNP data and by including spatial coordinates in the model-based Bayesian Geneland algorithm we were able to cluster individuals into groups that correspond to previously observed population structure. This demonstrates the benefit of including geographic coordinates to increase the power of inferring clusters in the presence of low genetic differentiation. By simple simulation studies, we also show the importance of estimating the size and effect of genotype errors when lower quality DNA is used.

## Methods

### Samples

In total, 2,044 anonymized samples from Sweden were genotyped. They were collected through the Swedish newborn screening registry as blood on filter paper. The sample set represents all newborns in Sweden from one week in December 2003, with 89 extra samples from the northern part of Sweden. Thus, the samples include native Swedes as well as immigrants. Sample collection and DNA extraction details are described elsewhere [19]. The samples were divided into counties and regions based on the information of birth hospital.

DNA from 627 unrelated Finnish male blood donors (465 for the STR analyses) who had given informed consent were collected through the Finnish Red Cross. The subjects represented a single generation with ages between 40 and 55 years, and were considered eligible when the birthplaces of their four grandparents clustered in the same geographical area, mostly in the same province of Finland.

Geographical coordinates for the Swedish birth hospitals and Finnish counties were identified using Google Earth v4.2. The Swedish county coordinates were calculated as the average coordinates weighted according to the number of samples over all birth hospitals in each county. For the Finnish counties, the coordinates of the geographical centre of the whole county were used, except for the Mantel tests where the coordinates were calculated as the average of individual coordinates of the samples representing each county. Individual coordinates of the Finnish samples were calculated as an average of the coordinates of their grandparental places of birth from Google Maps.

**Figure 1:**
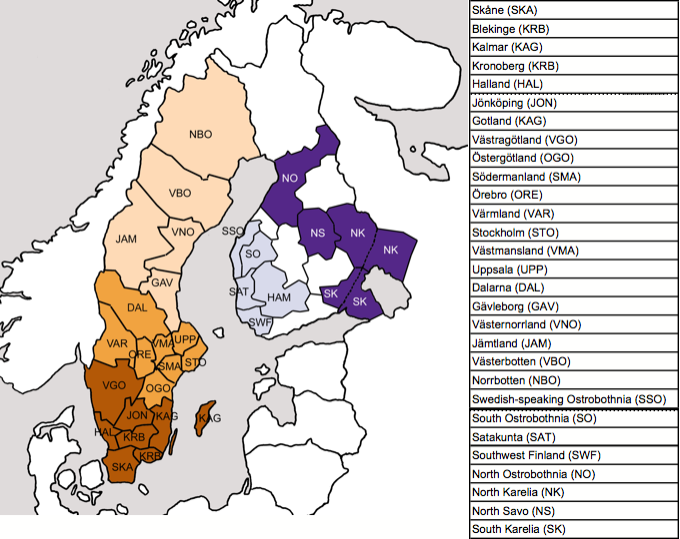
**Geographic location of the Swedish and Finnish counties**. The division of counties into five regions is denoted by their colour. Sample sizes are given in Additional file 1.

A map of the locations of the Finnish and Swedish counties is presented in Figure 1 [for sample sizes see Additional file 1]. For county-level analyses, the Swedish counties of Gotland and Kalmar, as well as Kronoberg and Blekinge were pooled in order to reach adequate sample sizes, and 13 Finnish samples were excluded from the analyses due to lacking county-level information of origin. Since the cities of Malmö and Gothenburg harbour large immigrant populations (in 2003, 33% and 26% of inhabitants had a foreign background, respectively; Statistics Sweden, ), some analyses, as indicated in the text, were performed also on a data set where these cities were excluded, in order to investigate whether the large percentage of immigrants affected the results.

The study was approved by the ethics committees of Karolinska Institutet, Stockholm and the Finnish Red Cross.

### Genotyping

Prior to genotyping, 30 μl (15–30 ng) of each extracted DNA from the Swedish Guthrie cards was amplified using improved primer preamplification (I-PEP-L). Three μl of each whole genome amplified DNA sample was used for SNP genotyping. Of the Finnish samples, 10 ng of genomic DNA was used for both SNP and STR genotyping. 43 SNPs, originally designed for zygosity testing [18], were assayed using the Sequenom MALDI-TOF platform and the iPLEX Gold chemistry (Sequenom, San Diego, California, USA). In order to minimize possible batch effects, the SNP genotyping was performed in parallel for both sample sets using the same primer and reagent mixtures. The Spectro Typer 3.4 software was used for automatic allele calling with two persons independently checking the genotype clusters. 31 STRs that were known to have rare alleles (frequency < 5%) in the Finnish population (Finnish Genome Center, unpublished data) were genotyped on a MegaBACE 1000 96-capillary electrophoresis instrument and called using the Genetic Profiler 1.1 software (Amersham Biosciences, Sunnyvale, California, USA). The genotypes were checked manually twice, blind to province information.

Sample, SNP and STR marker specific data are summarized in Additional files 1, 2 and 3, respectively. SNP-specific call and error rates are reported in Additional file 4. Samples with more than 20% missing data (445/2044 Swedish and 55/627 Finnish samples in the SNP data set, and 25/465 samples in the STR data set), X-linked markers (4 SNPs) and markers with more than 20% missing data in Finland or Sweden (5 SNPs, 1 STR) were excluded from the final data set. SNP genotyping error was estimated by 356 Swedish samples and 89 Finnish samples that were genotyped twice for each SNP marker. The whole genome amplification error rate was estimated by genotyping a set of 89 Swedish samples that had been whole genome amplified in two independent WGA reactions. The error rate for STR genotyping was estimated to be 0.025%, based on 1 observed discrepancy among 4,014 STR genotypes obtained in duplicate from independent MegaBACE runs.

Data extractions, filtering and calculation of basic summary statistics as well as genotype reports for PowerMarker and Geneland were done in Qlikview 7.5 (Qliktech, Sweden). Summary statistics and HWE calculations were performed in PowerMarker v3.5 [20]. PowerMarker and CONVERT v1.31 (Glaubitz JC, unpublished) were used for exporting genotype reports compatible with STRUCTURE, Arlequin, and PCA analysis.

### Analysis of molecular variance (AMOVA)

The genetic variation in Sweden and Finland was hierarchically partitioned into measures of genetic variation in individual, county, region and country levels by using F-statistics as implemented in R package Hierfstat [21] and Arlequin v3.1 [22] for F_individual/country _and F_individual/total_. We also performed an analysis of molecular variance (AMOVA) within and between countries, regions and counties using Arlequin v3.1. The significance levels for F-statistics and AMOVA results were based on 10,000 replications and 20,000 permutations, respectively.

### Isolation by distance (IBD)

Isolation by distance was investigated within and between Finland and Sweden by comparing matrices of pairwise F-statistics and great circle distances between counties using a Mantel test. The pairwise great circle distances were calculated in R package fields [23], and the pairwise F-statistics in Arlequin v3.1. The Mantel test was performed in R package ade4 [24], with the p-values based on 10,000 permutations.

### Identity by descent (IBS)

Population diversity was estimated as the identity by state between all the pairs of individuals within the countries and regions in R package GenABEL 1.3–5 [25]. The statistical significance of the differences between the IBS distributions of countries or regions was tested with a Mann-Whitney U test in R [26].

### Marker variance

The mean of marker variances was calculated from the Finnish dataset for SNP and STR markers in R [26].

### Principal components analysis (PCA)

Data were summarized in R [26] with principal component analysis on a covariance matrix of SNP and STR allele frequencies calculated over counties and regions for Finland and Sweden separately and combined.

### Chi-square tests

The allele frequency distributions between counties and between regions within Sweden and Finland were compared for each marker using a chi-square test. Nominal significancies were calculated in the chi-square test of R based on 100,000 replications. The overall significance of the findings of the multiple tests (34 for SNPs, 30 for STRs) was assessed by calculating the false discovery rate (FDR) as the ratio of expected to observed number of significants at the p < 0.01 level.

### Simulations of genotyping error and hidden population structure

The potential effect of genotyping errors and hidden population structure on the heterozygote deficiency and the HWE deviations observed in the Swedish dataset was studied with simulations. Genotypes of Swedish, European and non-European individuals were simulated assuming Hardy-Weinberg equilibrium within each group. Swedish and European allele frequencies were based on the Swedish and Finnish frequencies in our data, respectively; the non-European frequencies were based on HapMap data (YRI and CHB, HapMap data release #21). The effect of hidden population structure was then modelled by mixing the Swedish with 0–20% of either European or non-European individuals to form a sample of 1,599 individuals (corresponding to the size of our Swedish dataset). Genotyping errors were imposed on their genotypes, according to the whole-genome amplification error rates observed in the Swedish data [see Additional file 4]. The errors were either random or non-random in direction: in random errors, a homozygous genotype is called as a heterozygote and a heterozygote as either homozygote; in non-random errors, a heterozygote is called as a homozygote but homozygotes are called correctly, mimicking the phenomenon of allelic dropout where one of the heterozygote alleles fails to amplify. The total inbreeding coefficient F_IT _(corresponding to F_individual/country _of the real data) and the number of markers in Hardy-Weinberg disequilibrium (with p < 0.05 in a χ^2 ^test) were calculated from 1,000 such samples for each combination of genotyping error type and degree of hidden structure.

### Model-based clustering using Geneland

Clusters of individuals were inferred with the software Geneland [27-29]. This software implements an algorithm attempting to cluster samples on the basis of both genetic and geographic information. The geographic information is accounted for at the Bayesian prior level in such a way that clusters corresponding to spatially organized groups are considered more likely than those corresponding to completely random spatial patterns. The benefit of using a spatial prior (presumably more informative than a non-spatial prior) is to get more accurate inferences and to explicitly infer the spatial borders between inferred clusters.

Five datasets were studied: SNPs from the total data, Finland, Sweden with and without the cities of Malmö and Gothenburg, and STRs from Finland. We used the correlated frequency model treating the number of clusters as unknown. This was made possible through a substantial model improvement of the algorithm, which will soon be released as Geneland 3.0.0. We placed an independent Gamma prior on the drift coefficients with parameters (1,20). Geneland was then run 50 times for each dataset with 100,000 iterations and a burnin of 60,000 iterations. The runs were then sorted according to their mean posterior density and only the best ten runs were considered in the analysis.

### Model-based clustering using STRUCTURE

For comparison with Geneland, individuals were clustered with the Structure algorithm v2.2 [10,12] that does not use a spatial prior. Structure was run using a minimum of 10,000 burnins and iterations under both the non-admixture and admixture models assuming either correlated or non-correlated allele frequencies for values of K ranging from 1 to 5. Each parameter combination was run 5 times for each of the datasets described in the previous section to check the consistency of the clustering results.

## Results and discussion

### Observed east-western duality within Finland

**Figure 2:**
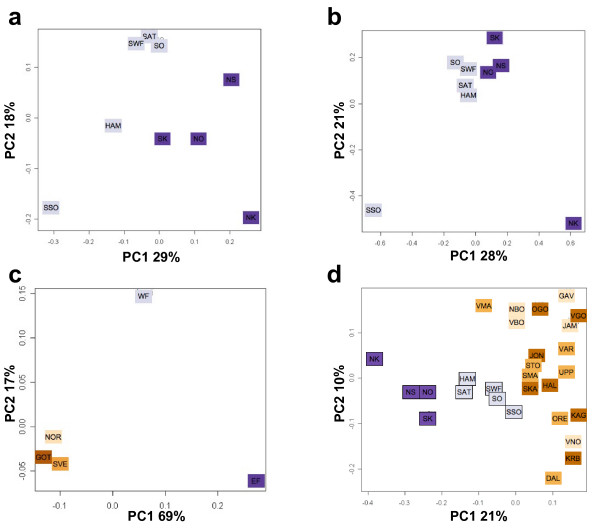
**Principal component analysis**. The principal components were extracted from covariance matrices based on frequencies of A) SNP minor alleles in Finnish counties, B) STR alleles in Finnish counties, C) SNP minor alleles in Finnish and Swedish regions and D) SNP minor alleles in Finnish and Swedish counties. The proportion of variance explained by each PC is shown on the axis. Abbreviations: Götaland (GOT), Svealand (SVE), Norrland (NOR), Western Finland (WF), Eastern Finland (EF); county abbreviations as in Figure 1.

**Figure 3:**
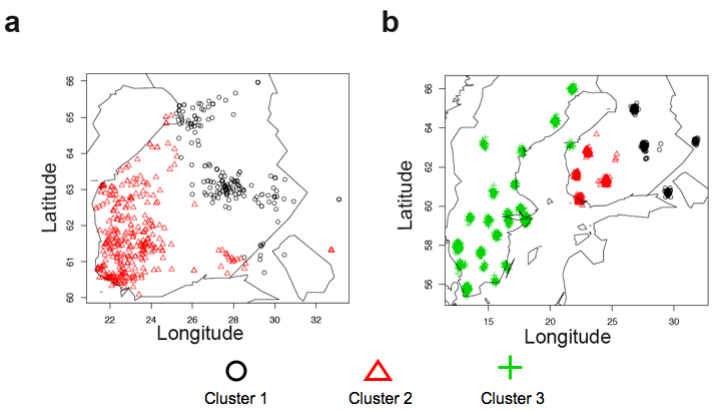
**Geneland clustering results**. The most likely cluster membership according to the Geneland algorithm using geographic coordinates as a prior and assuming correlated allele frequencies and no admixture between populations. A) Individual coordinates were used for the within-Finland analysis and B) county coordinates for the joint analysis between Sweden and Finland.

Out of an initial 627 individual DNA samples genotyped for the SNPs, 572 passed our quality criteria. This equals 90% of samples from Eastern Finland and 92% from Western Finland [see Additional file 1]. Within Finland, the Eastern and Western regions accounted for a small but significant portion (0.42%, p < 0.0001) of the genetic variation (Table 1); when the genetic structure was analysed between four hierarchical levels (Table 2), both F_region/country _and F_county/region _were significant (p < 0.01). This could suggest that in these subpopulations, genetic drift has had a greater impact than migration. The Mantel test for isolation by distance was significant (r = 0.32, p = 0.046; however, the exact significance seemed sensitive to the choice of county coordinates), indicating at least some clinal pattern of genetic variation within Finland. In the chi-square test, 6 SNPs (FDR = 0.06) showed significance between regions and 2 SNPs (FDR = 0.17) between counties at p < 0.01 level (see Additional file 5). The mean IBS was higher in Eastern than in Western Finland (0.656 and 0.649, respectively; p < 10^-66^), indicating higher homogeneity in the East. The first two components in the PCA loosely separated the eastern counties from the western (Figure 2A). The model-based Bayesian clustering algorithm implemented in Geneland inferred two clusters that corresponded, with the exception of a few samples, to East and West (Figure 3A). It is noteworthy, though, that the border between these two clusters runs somewhat further east than the regional division into East Finland and West Finland used in our data. The results are in agreement with previous studies that have identified a genetic border between the eastern and western parts of Finland that roughly coincides with several historical and anthropological borders as well as with regional differences in disease incidence [6].

**Table 1:** Results of the analysis of molecular variance (AMOVA) for SNP data indicating the distribution of genetic variation (in %) into the different hierarchical levels.

Dataset Subpopulation^a^		Total data county	Finland county	Finland region	Sweden county	Sweden region
subpopulation/total	%	0.43	0.42	0.42	0.15	0.03
	p	< 0.0001	ns	< 0.0001	ns	ns
individual/subpopulation	%	1.9	-0.53	-0.37	2.54	2.65
	p	< 0.0001	ns	ns	< 0.0001	< 0.0001
within individuals	%	97.68	100.11	99.96	97.32	97.32
	p	< 0.0001	ns	ns	< 0.0001	< 0.0001

The significance levels for the variance components were based on 20,000 permutations. ns = nonsignificant.

^a ^denotes the intermediate level of hierarchy used in the analysis

**Table 2:** F-statistics and their significances for the total data and Sweden and Finland separately.

F-statistic		Total data	Finland SNP	Finland STR	Sweden
F_country/total_	F	0.00371	-	-	-
	p	ns			
F_region/country_	F	0.00057	0.00323	0.00216	-0.00016
	p	< 0.01	< 0.01	< 0.05	ns
F_county/region_	F	0.00177	0.00236	0.00119	0.00157
	p	< 0.01	< 0.01	ns	ns
F_individual/county_	F	0.01734	-0.00529	-0.00022	0.02542
	p	< 0.0001	ns	ns	< 0.0001
F_individual/country_	F	0.01905	-0.00108	0.00209	0.02680
	p	< 0.0001	ns	ns	< 0.0001
F_individual/total_	F	0.02320	-	-	-
	p	< 0.0001			

The significance levels for F-statistics were based on 10,000 bootstraps. ns = nonsignificant.

In the STR dataset, 440 of the 465 samples (95%; 94% from east and 96% from west) passed our quality control. The division into Eastern and Western Finland accounted for a significant F_region/country _(Table 2), and the chi-square test showed 10 (FDR = 0.03) and 6 (FDR = 0.05) significants (p < 0.01) in the region and county level analyses, respectively [see Additional file 6]. Conversely, the Geneland algorithm inferred only a single cluster (data not shown), suggesting that the regional substructure was too weak to be detected even with the aid of the spatial prior, possibly due to lower sample size. The Mantel test for IBD was not significant (r = 0.01, p = 0.49). The first PC showed an east-west gradient and the second PC separated the two counties with smallest sample size from the others (Figure 2B). Although the F-statistic and Geneland analyses suggested a weaker structure in the STR than SNP data, the marker variances of the two datasets were rather similar (mean 0.133 for STRs and 0.136 for SNPs), and the numbers of chi-square significants were higher in STR than in SNP data [see Additional file 5 and 6], likely due to the larger number of alleles.

### No observed substructure but increased homozygosity in Sweden

A large portion, 445 out of 2,044, of the Swedish samples failed our quality controls [see Additional file 1], as expected due to the low DNA quality and the whole genome amplification. However, the amount of missing data was geographically non-differential, corresponding to 23%, 21% and 20% of samples from Götaland, Svealand and Norrland, respectively. The genetic variation between counties and regions in Sweden was non-significant and explained 0.15% and 0.03% respectively of the total variation (Table 1). F_individual/country _and F_individual/county _were significantly inflated (0.027 and 0.025 respectively, P < 0.0001, Table 2), and variation among individuals within counties and regions explained 2.5%.2.7% of the total variation (Table 1). IBS distributions differed significantly between regions (mean for Norrland 0.637, Svealand 0.639, Götaland 0.640; p < 10^-7 ^in all pairwise comparisons). The chi-square analysis [see Additional file 5] showed excess significants only between the counties (3 significants with p < 0.01, FDR = 0.11; 0 significants between regions), and some of this excess could be due to the genotyping errors.

The Mantel test for isolation by distance was non-significant (r = -0.014, p = 0.54), and the PCA lacked clear geographical patterns (data not shown), suggesting the absence of strong substructure within Sweden. However, many of the most deviating populations showed signs of drift also in the mtDNA and Y-chromosomal analysis (Lappalainen et al. 2008, submitted). Geneland consistently inferred a single cluster, regardless of inclusion or exclusion of the cities of Gothenburg and Malmö (data not shown). This suggests that higher levels of immigrant populations do not affect the observed results. As Geneland is able to account for null alleles [29], the presence of genotyping errors should not have a significant effect on the performance of the algorithm.

Despite the lack of clear substructure within Sweden, the Swedish dataset showed a heterozygote deficit (in terms of inflated F_individual/country _and F_individual/county_), and deviations from HWE in several markers [see Additional file 4]. It is known that the whole genome amplification or low-grade DNA can lead to allelic imbalances with a heterozygote deficit [30,31]. On the other hand, the observed deviations might be caused by hidden population structure, since our sampling includes both native Swedes and immigrants without any data on ethnicity.

**Figure 4:**
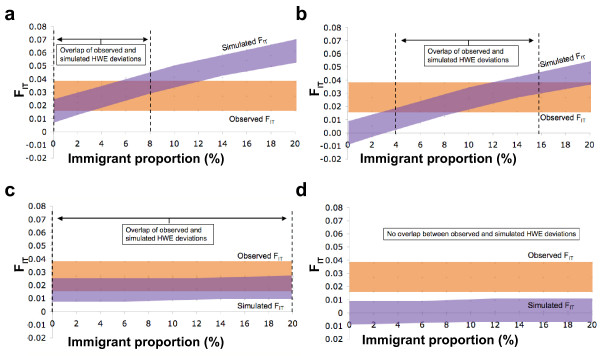
**Simulations of genotyping error and hidden population structure in Sweden**. The simulated effect of genotyping error and hidden population structure on the total fixation index F_individual/country _(F_IT_) and the number of markers deviating from HWE in the Swedish data. The 95% confidence bounds are based on 1,000 simulations. A) Non-random error and non-European substructure, B) random error and non-European substructure, C) non-random error and European substructure, D) random error and European substructure.

To estimate the relative role of these factors, we performed a simulation modeling the effect of immigration (European or non-European) or genotyping error (random or non-random) on F-statistic inflation and HWE deviations. The results (Figure 4) showed that random error and European immigration, either alone or in combination, could not account for the observed amount of deviations (Figure 4D), and that the observations fit best with a combination of both genotyping error and non-European ancestry (4% when the error is non-random and 10% when random) (Figure 4A–B). These levels of immigration correspond well to the situation in Sweden, where in 2003 approximately 16% of the inhabitants had a foreign background [see Additional file 7], and of them about 40% were non-Europeans (Statistics Sweden, ).

In the simulations, however, the level of genotyping error may be underestimated, since if the error is heterozygote-specific, the rates should be estimated relative to the number of heterozygotes in each marker. This would approximately double the error rates, in which case the non-random genotyping error could on its own explain the observed degree of deviations (data not shown). Whether or not these factors could mask a modest population structure remains unclear, but this kind of an error estimation approach would certainly be useful in contexts where it is known that the quality of the DNA may lead to informatively missing genotypes and consequent false positive associations [32,33].

### Genetic differences between Swedes and Finns

In the total data, the hierarchical F-statistics were significant except for F_country/total _(Table 2), and the Mantel test for IBD was significant (r = 0.39, p < 0.0003). The mean IBS was significantly lower in Sweden than in Finland (0.641 and 0.650, respectively; p << 10^-100^), indicating higher heterogeneity in Sweden. The principal component analysis clearly separated the Finnish regions and Eastern and Western counties from the Swedish as well as the Finnish regions and counties from each other (Figure 2C and 2D). Geneland showed three clusters (Figure 3B), roughly corresponding to Sweden, Eastern Finland and Western Finland. Thus, Geneland was able to correctly identify the country of origin of the individuals despite the lower quality of the Swedish data. Interestingly, the county-level PCA (Figure 2D) and Geneland (Figure 3B) placed the Finnish subpopulation of Swedish-speaking Ostrobothnia closest to Sweden. This minority population originates from the 13^th ^century, when Swedish settlers inhabited areas of coastal Finland [34]. Our result is in congruence with earlier studies where intermediate allele frequencies between Finns and Swedes have been observed in the Swedish speaking Finns [35].

### Comparison of methods for clustering of individuals

As described above, Geneland was able to infer two clusters in the Finns and three clusters in the total data when using a spatial prior. In contrast, the Structure software, which does not use a spatial prior, consistently found one cluster regardless of the data set and model used, as is expected from comparisons to earlier studies [36].

Geneland, in contrast with Structure, assumes that population membership is structured across space. If this assumption is correct, the power of inferring clusters increases; if the assumption is incorrect, it will lead to a loss of power but generally not to inference of spurious clusters (in the case of weak spatial organization, Geneland tends to perform like Structure in terms of inferred clusters [27]). Besides, in previous studies with similar goals it has been estimated that Structure needs a minimum of 65 to 100 random markers to separate continental groups and that the number of markers rather than samples is the most important parameter determining statistical power [13,37]. The differences between and within the neighbouring countries studied here are presumably smaller than those between continents and not large enough to be detected by Structure.

The detection of three clusters by Geneland versus one single cluster by Structure can thus be interpreted as an example of increased power in spatially structured populations. We however conjecture that on other human datasets, still with spatial structure but lower differentiation, Geneland would require a larger number of markers to uncover population structure.

From a methodological point of view it should be mentioned that PCA is mostly descriptive and performs no formal estimation or testing of the presence of groups. Also, even if PCA detects structure in the form of clusters, nothing can be said about the genetic features of these. In contrast, Geneland tries to find and estimate the number of clusters displaying HWE and linkage equilibrium. Also, since no population labels or predefined groups are used in the Geneland analyses, the results are more objective and should consequently be more representative of the real genetic substructure than when a clustering method is used on predefined populations, as in the PCA analysis of allele frequencies in this paper.

### Internal and external validity of the study

The difference in genotyping quality and sampling between the Finnish and Swedish samples may affect the external validity of the study, and the validity of comparisons between Sweden and Finland. The ancestry of the Finnish samples is well ascertained through grandparental places of birth, although the coverage of the central part of the country is suboptimal. Also, since the Finnish samples represent the rural communities, they probably underestimate the effect of recent migration within Finland. These factors might exaggerate the sharpness of the observed genetic border, and the true pattern of contemporary variation may be more clinal. For the Swedes, we only have information about the birth hospital of each individual. It is an imperfect proxy for home county, but should be highly correlated with it and therefore applicable for the purpose of investigating the modern population structure. However, these samples are not appropriate for drawing conclusions about the historical population structure since the sample set is affected by immigration and recent migration within Sweden. Likewise, the observed genotyping error probably contributes at least partly to the heterozygote deficit observed in Sweden, and while the error is geographically non-differential, it may obscure some existing substructure. This study therefore also emphasizes the importance of trying to estimate the effect, type and size of genotyping errors in population genetics [38,39].

Population genetic measures are affected by the marker discovery approach, as has been shown for both SNPs and the dominant amplified fragment length polymorphisms (AFLP) [40,41]. In our study, the SNPs have been chosen from the HapMap data to maximize the observed difference between individuals, and are thus more heterozygous than random SNPs [18,42]. This should increase their power to detect structure in our closely related populations [43]. However, our SNPs probably underestimate the differences between continents, which may have affected the simulations of immigrant contribution. The STR markers have been selected to have a high number of rare alleles within Finland, and in combination with the lower sample size, this can explain why the STR analyses appeared less efficient than SNPs in detecting substructure within Finland.

## Conclusion

Using 34 unlinked autosomal SNPs, we detected a small but significant structure within Finland, especially between the eastern and western region, supporting the previously documented east-west duality in Finland [3,4]. In the Swedish data we did not detect similar patterns, which suggests a lack of geographic population structure within the country, but can also be compatible with a modest substructure. The Swedish data showed a heterozygote deficiency, which led us to show that it is important to estimate the effect of genotyping error that may otherwise distort the conclusions drawn from the data. Our results from the Geneland algorithm demonstrate the benefit of including spatial information in clustering individuals according to their genetic similarity, particularly at low levels of differentiation. Although Geneland has successfully clustered individuals into groups with low or moderate F_ST_in ecological studies [44-46], to the best of our knowledge, this is the first time the algorithm has been used for human or SNP data.

## List of abbreviations

AIM: Ancestry informative marker; AMOVA: Analysis of molecular variance; FDR: False discovery rate; HWE: Hardy-Weinberg equilibrium; IBD: Isolation by distance; mtDNA: mitochondrial DNA; PCA: Principal components analysis; SNP: Single nucleotide polymorphism; STR: Short tandem repeat; WGA: Whole genome amplification.

## Authors' contributions

UH conceived and took part in designing the study, prepared the Swedish samples, did the SNP genotyping and simulation studies, participated in the statistical analyses and drafted the paper. ES participated in the preparation of the Finnish samples and drafting the paper, performed the STR genotyping, PCA and chi-square analyses, and participated in the other statistical analyses. TL participated in the preparation of the Finnish samples, drafting the paper, and the statistical analyses. GG performed the Geneland analyses and participated in the statistical analyses and drafting the paper. CML took part in designing the study. UvD provided the Swedish samples. PL took part in designing the study. JK took part in conceiving and designing the study. All authors reviewed the manuscript and accepted the final version.

## Supplementary Material

Additional file 1 The number of samples from each county and region in Finland and Sweden. The sample sizes both before and after quality control are given for both the SNP and the STR analysis. Click here for file

Additional file 2 SNP marker information and allele frequencies for data from pooled Swedes and Finns, Swedes, Finns, and HapMap populations. The reference alleles for the frequency calculations are based on the minor allele frequencies in the pooled Finnish and Swedish data. Click here for file

Additional file 3 Summary information of the STR data set of the Finnish samples. The HWE p-values are based on 10,000 permutations of the observed alleles into genotypes. Click here for file

Additional file 4 SNP genotyping error estimates based on replicate genotypes from 356 Swedish and 89 Finnish samples. The whole genome amplification error rate was estimated by genotyping a set of 89 Swedish samples that had been whole genome amplified in two independent WGA reactions. Hardy-Weinberg equilibrium p-values are based on a standard χ^2^-test. Click here for file

Additional file 5 The results of chi-square tests for the distribution of allele frequencies of SNP markers between the Finnish and Swedish counties and regions. The p-values are based on 100,000 permutations. Those with a nominal significance below 0.01 are bolded, with their numbers and the false discovery rate (FDR) summarized below. Click here for file

Additional file 6 The results of chi-square tests for the distribution of allele frequencies of STR markers between the Finnish counties and regions. The p-values are based on 100,000 permutations. Those with a nominal significance below 0.01 are bolded, with their numbers and the false discovery rate (FDR) summarized below. Click here for file

Additional file 7 Number of individuals with foreign background (born abroad or both parents born abroad) in Sweden in 2003, stratified by county and region. Click here for file

## References

[B1] Siiriäinen A, Helle K, Jansson T (2003). The Stone and Bronze Ages: In Helle K. Jansson T (eds): The Cambridge History of Scandinavia. The Cambridge History of Scandinavia.

[B2] Lindqvist H (2006). A history of Sweden.

[B3] Lappalainen T, Koivumaki S, Salmela E, Huoponen K, Sistonen P, Savontaus ML, Lahermo P (2006). Regional differences among the Finns: a Y-chromosomal perspective. Gene.

[B4] Kittles RA, Perola M, Peltonen L, Bergen AW, Aragon RA, Virkkunen M, Linnoila M, Goldman D, Long JC (1998). Dual origins of Finns revealed by Y chromosome haplotype variation. Am J Hum Genet.

[B5] Norio R (2003). The Finnish Disease Heritage III: the individual diseases. Hum Genet.

[B6] Norio R (2003). Finnish Disease Heritage II: population prehistory and genetic roots of Finns. Hum Genet.

[B7] Norio R (2003). Finnish Disease Heritage I: characteristics, causes, background. Hum Genet.

[B8] Karlsson AO, Wallerstrom T, Gotherstrom A, Holmlund G (2006). Y-chromosome diversity in Sweden - a long-time perspective. Eur J Hum Genet.

[B9] Einarsdottir E, Egerbladh I, Beckman L, Holmberg D, Escher SA (2007). The genetic population structure of northern Sweden and its implications for mapping genetic diseases. Hereditas.

[B10] Pritchard JK, Rosenberg NA (1999). Use of unlinked genetic markers to detect population stratification in association studies. Am J Hum Genet.

[B11] Rosenberg NA, Pritchard JK, Weber JL, Cann HM, Kidd KK, Zhivotovsky LA, Feldman MW (2002). Genetic structure of human populations. Science.

[B12] Falush D, Stephens M, Pritchard JK (2003). Inference of population structure using multilocus genotype data: linked loci and correlated allele frequencies. Genetics.

[B13] Turakulov R, Easteal S (2003). Number of SNPS loci needed to detect population structure. Hum Hered.

[B14] Yang N, Li H, Criswell LA, Gregersen PK, Alarcon-Riquelme ME, Kittles R, Shigeta R, Silva G, Patel PI, Belmont JW, Seldin MF (2005). Examination of ancestry and ethnic affiliation using highly informative diallelic DNA markers: application to diverse and admixed populations and implications for clinical epidemiology and forensic medicine. Hum Genet.

[B15] Enoch MA, Shen PH, Xu K, Hodgkinson C, Goldman D (2006). Using ancestry-informative markers to define populations and detect population stratification. J Psychopharmacol.

[B16] Seldin MF, Price AL (2008). Application of ancestry informative markers to association studies in European Americans. PLoS Genet.

[B17] Allocco DJ, Song Q, Gibbons GH, Ramoni MF, Kohane IS (2007). Geography and genography: prediction of continental origin using randomly selected single nucleotide polymorphisms. BMC Genomics.

[B18] Hannelius U, Gherman L, Makela VV, Lindstedt A, Zucchelli M, Lagerberg C, Tybring G, Kere J, Lindgren CM (2007). Large-scale zygosity testing using single nucleotide polymorphisms. Twin Res Hum Genet.

[B19] Hannelius U, Lindgren CM, Melen E, Malmberg A, von Dobeln U, Kere J (2005). Phenylketonuria screening registry as a resource for population genetic studies. J Med Genet.

[B20] Liu K, Muse SV (2005). PowerMarker: an integrated analysis environment for genetic marker analysis. Bioinformatics.

[B21] Goudet J (2005). Hierfstat, a package for R to compute and test hierarchical F-statistics.. Molecular Ecology Notes.

[B22] Laval LG, Schneider S, Excoffier (2005). Arlequin ver. 3.0: An integrated software package for population genetics data analysis.. Bioinformatics Online.

[B23] Nychka D (2007). Fields: Tools for spatial data. R package version 4.1.. http://www.image.ucar.edu/GSP/Software/Fields.

[B24] Dray S, Dufour AB (2007). The ade4 package: implementing the duality diagram for ecologists.. Journal of Statistical Software.

[B25] Yurii Aulchenko MS (2008). GenABEL: genome-wide SNP association analysis. R package version 1.3-5..

[B26] R_Development_Core_Team (2008). R: A language and environment for statistical computing.

[B27] Guillot G, Estoup A, Mortier F, Cosson JF (2005). A spatial statistical model for landscape genetics. Genetics.

[B28] Excoffier L, Heckel G (2006). Computer programs for population genetics data analysis: a survival guide. Nat Rev Genet.

[B29] Guillot G, Santos F, Estoup A (2008). Analysing georeferenced population genetics data with Geneland: a new algorithm to deal with null alleles and a friendly graphical user interface. Bioinformatics.

[B30] Dixon LA, Dobbins AE, Pulker HK, Butler JM, Vallone PM, Coble MD, Parson W, Berger B, Grubwieser P, Mogensen HS, Morling N, Nielsen K, Sanchez JJ, Petkovski E, Carracedo A, Sanchez-Diz P, Ramos-Luis E, Brion M, Irwin JA, Just RS, Loreille O, Parsons TJ, Syndercombe-Court D, Schmitter H, Stradmann-Bellinghausen B, Bender K, Gill P (2006). Analysis of artificially degraded DNA using STRs and SNPs--results of a collaborative European (EDNAP) exercise. Forensic Sci Int.

[B31] Lovmar L, Fredriksson M, Liljedahl U, Sigurdsson S, Syvanen AC (2003). Quantitative evaluation by minisequencing and microarrays reveals accurate multiplexed SNP genotyping of whole genome amplified DNA. Nucleic Acids Res.

[B32] Clayton DG, Walker NM, Smyth DJ, Pask R, Cooper JD, Maier LM, Smink LJ, Lam AC, Ovington NR, Stevens HE, Nutland S, Howson JM, Faham M, Moorhead M, Jones HB, Falkowski M, Hardenbol P, Willis TD, Todd JA (2005). Population structure, differential bias and genomic control in a large-scale, case-control association study. Nat Genet.

[B33] Plagnol V, Cooper JD, Todd JA, Clayton DG (2007). A method to address differential bias in genotyping in large-scale association studies. PLoS Genet.

[B34] Pitkänen K (1994). Suomen väestön historialliset kehityslinjat (Historical trends in the development of the Finnish population). In Koskinen S, Martelin T, Notkola IL, Notkola V, Pitkänen K (eds.): Suomen väestö (The population of Finland)..

[B35] Virtaranta-Knowles K, Sistonen P, Nevanlinna HR (1991). A population genetic study in Finland: comparison of the Finnish- and Swedish-speaking populations. Hum Hered.

[B36] Steffens M, Lamina C, Illig T, Bettecken T, Vogler R, Entz P, Suk EK, Toliat MR, Klopp N, Caliebe A, Konig IR, Kohler K, Ludemann J, Diaz Lacava A, Fimmers R, Lichtner P, Ziegler A, Wolf A, Krawczak M, Nurnberg P, Hampe J, Schreiber S, Meitinger T, Wichmann HE, Roeder K, Wienker TF, Baur MP (2006). SNP-based analysis of genetic substructure in the German population. Hum Hered.

[B37] Rosenberg NA, Mahajan S, Ramachandran S, Zhao C, Pritchard JK, Feldman MW (2005). Clines, clusters, and the effect of study design on the inference of human population structure. PLoS Genet.

[B38] Bonin A, Bellemain E, Bronken Eidesen P, Pompanon F, Brochmann C, Taberlet P (2004). How to track and assess genotyping errors in population genetics studies. Mol Ecol.

[B39] Pompanon F, Bonin A, Bellemain E, Taberlet P (2005). Genotyping errors: causes, consequences and solutions. Nat Rev Genet.

[B40] Rosenblum EB, Novembre J (2007). Ascertainment bias in spatially structured populations: a case study in the eastern fence lizard. J Hered.

[B41] Foll M, Beaumont MA, Gaggiotti OE (2008). An Approximate Bayesian Computation approach to overcome biases that arise when using AFLP markers to study population structure. Genetics.

[B42] Petkovski E, Keyser-Tracqui C, Hienne R, Ludes B (2005). SNPs and MALDI-TOF MS: tools for DNA typing in forensic paternity testing and anthropology. J Forensic Sci.

[B43] Clark AG, Hubisz MJ, Bustamante CD, Williamson SH, Nielsen R (2005). Ascertainment bias in studies of human genome-wide polymorphism. Genome Res.

[B44] Coulon A, Guillot G, Cosson JF, Angibault JM, Aulagnier S, Cargnelutti B, Galan M, Hewison AJ (2006). Genetic structure is influenced by landscape features: empirical evidence from a roe deer population. Mol Ecol.

[B45] Latch EK, Scognamillo DG, Fike JA, Chamberlain MJ, Rhodes OE (2008). Deciphering Ecological Barriers to North American River Otter (*Lontra canadensis*) Gene Flow in the Louisiana Landscape. J Hered.

[B46] Rowe G, Beebee TJ (2007). Defining population boundaries: use of three Bayesian approaches with microsatellite data from British natterjack toads (*Bufo calamita*). Mol Ecol.

